# Brain and Blood Transcriptome‐Wide Association Studies Identify Three Novel Genes Associated with Cognitive Resilience

**DOI:** 10.1002/alz.093123

**Published:** 2025-01-03

**Authors:** Makaela Mews, Adam C. Naj, Jennifer Elizabeth Below, Logan C. Dumitrescu, Timothy J. Hohman, Anthony J. Griswold, William S. Bush

**Affiliations:** ^1^ System Biology & Bioinformatics; Department of Nutrition; Case Western Reserve University, Cleveland, OH USA; ^2^ Penn Neurodegeneration Genomics Center, Department of Pathology and Laboratory Medicine, Perelman School of Medicine, University of Pennsylvania, Philadelphia, PA USA; ^3^ University of Pennsylvania, Perelman School of Medicine, Department of Biostatistics and Epidemiology/Center for Clinical Epidemiology and Biostatistics, Philadelphia, PA USA; ^4^ Vanderbilt Genetics Institute and Division of Genetic Medicine, Vanderbilt University Medical Center, Nashville, TN USA; ^5^ Vanderbilt Memory & Alzheimer’s Center and Vanderbilt Genetics Institute, Vanderbilt University Medical Center, Nashville, TN USA; ^6^ John P. Hussman Institute for Human Genomics, University of Miami Miller School of Medicine, Miami, FL, USA, Miami, FL USA; ^7^ Dr. John T. Macdonald Foundation Department of Human Genetics, University of Miami Miller School of Medicine, Miami, FL USA; ^8^ Cleveland Institute for Computational Biology, Department of Population and Quantitative Health Sciences, Case Western Reserve University, Cleveland, OH USA

## Abstract

**Background:**

While there are numerous genome‐wide association studies (GWAS) assessing the genetic basis of Alzheimer’s disease (AD), there are far fewer studies examining genetic factors for cognitive and global resilience to AD neuropathology. By focusing on a gene‐level rather than a single‐variant basis, transcriptome‐wide association studies (TWAS) have increased statistical power relative to GWAS and can assess the role of genetically regulated gene expression in AD resilience. We leveraged the largest available cis‐eQTL meta‐analysis summary statistics from brain tissue (MetaBrain Brain‐Cortex; N = 2,547) and whole blood (eQTLGen; N = 31,684) and applied them to the largest cognitive and global resilience to AD neuropathology summary statistics from Dumitrescu et al. 2020 (N = 5,108; Cognitively Unimpaired(CU) = 3,820) to identify novel resilience genes using the OTTERS TWAS pipeline.

**Method:**

We modified the OTTERS approach (Dai et al. 2023) to only include genes with concordant effect size directions across the five TWAS methods it implements. We used the 1000 Genomes high‐coverage dataset as a LD reference, and characterized genes as novel by excluding regions that overlapped a 1MB window around the previously identified GWAS hits from the GWAS catalog. We also validated our hits using one‐tailed T‐tests applied to RNA‐seq expression data from an independent NHW whole‐blood dataset (MAGENTA: AD = 119; Controls = 117) and a partially independent brain RNA microarray dataset called KRONOSII that was curated for high AD pathology and low secondary pathology (AD = 168; Controls = 177).

**Result:**

Within blood, we identified and validated one gene (*LCLAT1*, p = 1.97e‐10) where the expression was positively associated with cognitive resilience in a CU subset and we confirmed AD cases had significantly lower expression relative to controls. Within brain, we identified and validated two genes that were negatively associated with all resilience phenotypes (*SNX7*, p = 8.05e‐08) or resilience using CU subsets (*FAM117B*, p = 2.34e‐13) and we validated AD cases had significantly higher expression relative to controls. Notably, SNX7 is a member of the sorting nexin protein family which have been implicated in APP trafficking and degradation.

**Conclusion:**

By combining multiple large scale eQTL summary statistics with resiliency GWAS results, we have identified multiple new gene associations to cognitive and global resilience to AD.

